# Orthogonal colloidal quantum dot inks enable efficient multilayer optoelectronic devices

**DOI:** 10.1038/s41467-020-18655-7

**Published:** 2020-09-23

**Authors:** Seungjin Lee, Min-Jae Choi, Geetu Sharma, Margherita Biondi, Bin Chen, Se-Woong Baek, Amin Morteza Najarian, Maral Vafaie, Joshua Wicks, Laxmi Kishore Sagar, Sjoerd Hoogland, F. Pelayo García de Arquer, Oleksandr Voznyy, Edward H. Sargent

**Affiliations:** 1 Department of Electrical and Computer Engineering, University of Toronto, 35 St George Street, Toronto, ON M5S 1A4 Canada; 2 Department of Physical and Environmental Sciences, University of Toronto Scarborough, Scarborough, ON M1C 1A4 Canada; 3 Present Address: Department of Chemical and Biochemical Engineering, Dongguk University, Seoul, 04620 Republic of Korea; 4 Present Address: Department of Chemical and Biological Engineering, Korea University, 145 Anam-Ro, Seongbuk-Gu, Seoul, 02841 Republic of Korea

**Keywords:** Solar cells, Quantum dots

## Abstract

Surface ligands enable control over the dispersibility of colloidal quantum dots (CQDs) via steric and electrostatic stabilization. Today’s device-grade CQD inks have consistently relied on highly polar solvents: this enables facile single-step deposition of multi-hundred-nanometer-thick CQD films; but it prevents the realization of CQD film stacks made up of CQDs having different compositions, since polar solvents redisperse underlying films. Here we introduce aromatic ligands to achieve process-orthogonal CQD inks, and enable thereby multifunctional multilayer CQD solids. We explore the effect of the anchoring group of the aromatic ligand on the solubility of CQD inks in weakly-polar solvents, and find that a judicious selection of the anchoring group induces a dipole that provides additional CQD-solvent interactions. This enables colloidal stability without relying on bulky insulating ligands. We showcase the benefit of this ink as the hole transport layer in CQD optoelectronics, achieving an external quantum efficiency of 84% at 1210 nm.

## Abstract (web summary)

The realisation of film made up of different compositions using colloidal QD inks remains a challenge because of redispersing of underlying films by polar solvents. Here, the authors introduce aromatic ligands to achieve QD inks in weakly-polar solvents that enable fabrication of multi-compositional films.

## Introduction

Colloidal quantum dots (CQDs) have been explored in optoelectronic applications such as photovoltaics (PVs)^1–3^, photodetectors^4–6^, light-emitting diodes^7–9^, and lasers^10,11^, a testament to their facile bandgap tuning and solution processing. Lead chalcogenide CQDs have the advantage of wide-ranging bandgap tunability across the visible to the infrared (IR) (~0.6 eV), extending the PV and photodetector response beyond the c-Si bandgap (*E*_*g*_ = 1.1 eV). In light of their IR absorption, CQDs are promising materials for use in short-wavelength IR photodetectors^12^ and as back cells^13,14^ in tandems with Si (*E*_*g*_ = 1.1 eV) and perovskites (1.58–1.68 eV).

The synthesis of CQDs relies on the use of long insulating ligands such as oleic acid (OA) and oleylamine. For applications in optoelectronic devices, these must be replaced with shorter ligands to improve carrier transport in CQD films. Early device prototypes employed a layer-by-layer (LBL) deposition strategy, known as solid-state ligand exchange, to fabricate CQD films^1,15–18^. However, the LBL solid-state ligand exchange process is incompatible with scalable methods of manufacture such as roll-to-roll. Solid-state ligand exchanges also lead to inhomogeneous energy states in CQD films^19–23^.

Solution-phase ligand exchanges have been developed to overcome these issues^24–29^: they enable the formation of smooth and crack-free CQD films through a single, 100%-materials-utilization, deposition. High-quality CQD inks based on short ligands, such as lead halides^25–27^, acetate salts^26,27^, and thiols^3,28^, have been demonstrated.

These works have relied on the use of highly polar solvents. This approach stands in the way of the creation of multifunctional multilayer devices—structures in which the different layers incorporate distinct sizes and composition of quantum dots—since highly polar solvents disperse the prior layers. For this reason, today’s highest-performing CQD optoelectronic devices rely on a solid-state ligand exchange step^1,3,25–27^ to construct the final CQD hole transport layer (HTL) atop the main active layer.

These considerations mandate the development of orthogonal CQD inks that are sufficiently nonpolar to preserve the underlying layers. However, conventional nonpolar and low-polarity CQD inks have relied on long electrically insulating hydrophobic ligands since shorter ligands produce CQD aggregation in low-polarity solvents.

We reasoned that—since aromatic rings are known to be highly polarizable—a judicious selection of aromatic ligands, including of their anchoring group, would induce a dipole for increased interaction with a weakly polar solvent, enabling colloidal stability.

Here we report as a result a ligand strategy that enables CQD inks based on weakly polar solvents. Using a combination of experiment and computation, we explore the effect of the anchoring group of the ligand on the solubility of the CQD ink. We find that the stronger electron-withdrawing effect of the carboxyl group results in polarization of the aromatic ring relative to the thiol group, enabling benzoic acid (BA)-exchanged CQDs to be stabilized in chlorobenzene. In contrast, benzenethiol (BT)-exchanged CQDs aggregate and precipitate.

We deploy the weakly polar CQD inks to devise smooth and crack-free HTLs that exhibit reduced trap densities compared to conventional ethanedithiol (EDT)-exchanged HTLs reliant on solid-state ligand exchange. Single-step spin-coating with chlorobenzene enables the fabrication of HTLs without degradation of the CQD layer beneath, whereas the solid-state ligand exchange procedure for the deposition of the EDT-exchanged HTL leads to trap states in the underlying light-absorbing layer. We realize fully ink-based CQD PV, achieving an external quantum efficiency (EQE) of 84% at 1210 nm. This results in addition of 1.43% and 3.57% additional solar-power points atop those of silicon and perovskite solar cells, respectively.

## Results

### Synthesis of weakly polar CQD inks

Conventional nonpolar CQD inks rely on bulky ligands, leading to compromised charge transport. In a previous report, 4-methylbenzenethiol (4-CH_3_-BT)-exchanged PbS CQDs were stabilized in weakly polar solvents such as dichloromethane and dichlorobenzene^19^. However, the 4-CH_3_-BT-exchanged CQD films showed limited charge transport, resulting in low PV performance and a power conversion efficiency (PCE) of 1.34%. We sought therefore to use a shorter BT ligand to synthesize a weakly polar CQD ink without the methyl pending group required in previous works; however, the BT-exchanged PbS CQDs were substantially insoluble in weakly polar solvents including chloroform, dichloromethane, and chlorobenzene (Supplementary Fig. 1a).

**Fig. 1:**
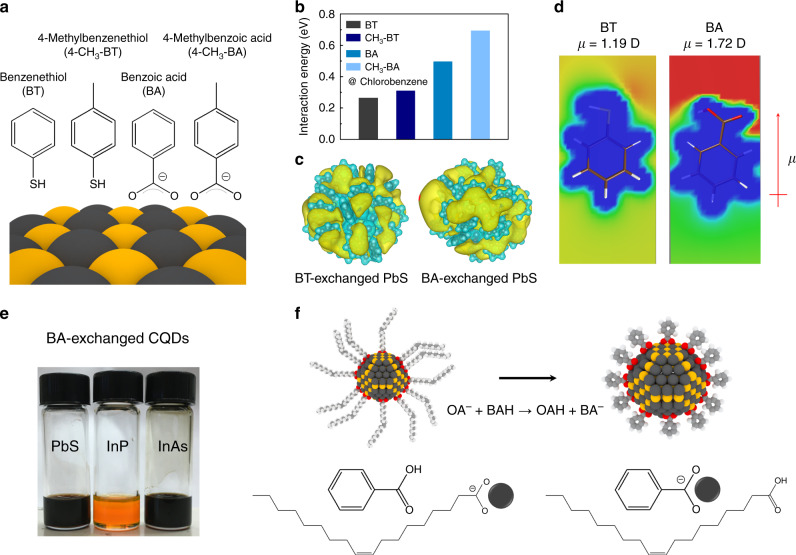
Orthogonal CQD inks. **a** Chemical structure of aromatic ligands. **b** Interaction energies of four different ligand-exchanged CQDs with chlorobenzene. **c** Electrostatic potentials of entire CQDs capped with BT and BA. **d** Electrostatic potentials of isolated BT and BA ligands. **e** Photograph showing the dispersibility of the BA-exchanged CQDs (PbS, InP/ZnSe/ZnS, and InAs) in chlorobenzene. **f** Schematic illustrations of the ligand exchange procedure.

Colloidal stabilization of CQDs requires a sufficient CQD-solvent interaction to overcome the CQD–CQD attraction. Given that aromatic molecules are highly polarizable, we reasoned that the anchoring group of ligands enables control over the polarization of a benzene ring, providing an additional means to enhance CQD-solvent interactions in the case when the solvent is weakly polar. We reasoned that the carboxyl group induce a stronger dipolar polarization of the aromatic ring compared to the thiol group^30^, and that this can enhance interactions of the CQD with a weakly polar solvent. For this reason, we sought to use BA ligands to synthesize CQD inks stabilized in weakly polar solvents (Fig. 1a) toward the ultimate goal of efficient charge transport in final CQD films. This allowed colloids in a variety of weakly polar solvents (Supplementary Fig. 1b) that included chlorobenzene, toluene, dichloromethane, chloroform, and anisole, without the need for an additional pending methyl group previously employed.

We carried out density functional theory (DFT) calculations to elucidate the effect of the ligand anchoring group and the pendant methyl group on the solubility of CQDs. We studied a series of four different aromatic ligands and calculated the interaction energies of exchanged CQDs with chlorobenzene solvent (Fig. 1b and Supplementary Table 1). The presence of a methyl group in the para position enhanced the interaction energy for both BT- and BA-functionalized CQDs, supporting previous findings that methyl group improves the solubility of CQDs in weakly polar solvents. Experimental results showed that without a methyl group, BT-exchanged CQDs are insoluble in chlorobenzene.

The BA-exchanged CQDs showed significantly higher interaction energy with chlorobenzene despite the fact that the outer surface of the ligand is identical—a finding consistent with the hypothesized polarization effect. The computed electrostatic potential of an entire CQD capped with ligands reveals larger pockets of positive potential. These pockets are available for interaction with the solvent dipole (Fig. 1c), and are consistent with the electrostatic potentials of isolated ligands (Fig. 1d). Experimentally, we found that—despite the improvement of solubility in the presence of one methyl group—4-CH_3_-BT-exchanged CQDs showed lower solubility than the BA-exchanged CQDs. The BA-exchanged CQDs remained stable as colloids, producing no observable aggregation following 6 months’ storage in air (Supplementary Fig. 2).

To challenge the versatility of the ligand exchange strategy, we sought to deploy it also on InAs and InP CQDs. We used different combinations of solvents/solvent mixtures depending on the CQDs since the dynamics of ligand exchange depend on the CQD surface chemistry. With optimal ligand exchange conditions for different type of CQD, we achieved stable BA-exchanged CQDs in chlorobenzene (Fig. 1e).

We depict in Fig. 1f the aromatic ligand exchange to synthesize weakly polar CQD inks. The initial CQDs are capped with OA and dispersed in toluene. The aromatic ligands dissolved in an appropriate solvent are added dropwise to the CQD solution while stirring, leading to replacement of the bulky OA ligands. After ligand exchange, the OA byproduct and the residual ligands are removed using multiple purification steps, and the exchanged CQDs can be dispersed in a variety of solvents (see ligand exchange details in “Methods” section and Supplementary Table 2).

We performed Fourier transform infrared spectroscopy (FTIR) and found that, after ligand exchange with BT and BA, the linear alkane and alkene C–H stretches were no longer observed (Supplementary Fig. 3), indicating complete removal of OA from the CQD surface.

### Electrical and optical characterization of HTLs

**Fig. 2:**
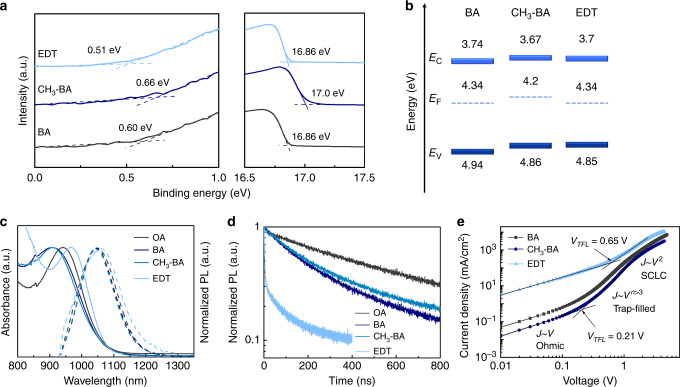
Characterization of HTLs. **a** UPS spectra of the BA-functionalized CQD films and the conventional EDT-exchanged film. **b** Energy levels of the BA-functionalized CQD films and the EDT-exchanged film. **c** Normalized absorption and PL spectra of CQD films before and after ligand exchange. **d** Time-resolved PL spectra of CQD films before and after ligand exchange. **e** *J–V* characteristics of HODs with different HTLs.

We used BA-functionalized PbS CQD inks (*E*_*g*_ = 1.3 eV) to fabricate CQD films as HTLs for solid-state ligand exchange-free CQD PVs. We performed ultraviolet photoelectron spectroscopy (UPS) measurements of the BA-functionalized CQD films and the conventional solid-state EDT-exchanged film to determine the Fermi level (*E*_*F*_) and valence band maximum (VBM) (Fig. 2a). The EDT-exchanged CQD film showed a more p-type character, consistent with the higher S/Pb atomic ratio resulting from the use of thiol ligands^3^. Figure 2b shows the energy level diagram of the CQDs exchanged with BA, 4-methylbenzoic acid (4-CH_3_-BA), and EDT, determined using UPS (Fig. 2a) and UV–vis absorption (Fig. 2c). The three different CQD films do not show a noticeable difference in their energy levels. In this work, we used PbS CQDs (*E*_*g*_ = 1.0 eV) as the main light-absorbing active layer. The VBMs (~4.9 eV) of the CQD HTLs are shallower than the VBM (5.2 eV) of the main CQD light-absorbing layer, enabling efficient hole extraction from the active layer. The high-lying conduction bands (~3.7 eV) of the three different CQD HTLs can block electrons from the active layer (conduction band ~4.2 eV), thereby suppressing bimolecular recombination at the HTL/active layer interface.

To characterize the optical properties of the CQD films exchanged with BA, 4-CH_3_-BA, and EDT, we carried out UV–vis absorption as well as steady-state and time-resolved photoluminescence (PL) spectroscopy (Fig. 2c, d). EDT-exchanged films exhibited bandtail PL in the wavelength region >1200 nm (Fig. 2c), indicating that the ligand exchange from OA to EDT creates trap states within the bandgap.

The BA-functionalized CQD films showed shorter PL lifetime than did OA-capped CQD films (Fig. 2d), which may be attributed to exciton dissociation via charge or energy transfer caused by the enhanced inter-dot coupling^31,32^. The EDT-exchanged film showed a substantially reduced PL lifetime compared to the BA-functionalized CQD films. We offer two possible explanations for this. One is that the shorter length of EDT ligands enhances inter-dot coupling, leading to faster exciton dissociation via charge or energy transfer. Another possibility is an increased density of trap states in the EDT-exchanged films functions as charge acceptors, reducing the PL decay time. Using an independent method—space-charge-limited current trap-filling studies of Fig. 2e—we found that the EDT-exchanged CQD films showed a 3.5× higher trap state density than did the BA-functionalized CQD films. We therefore offer that a significant contributor to the faster PL decay time in the EDT-exchanged CQD films can include the effect of trap states.

To investigate further the mobility and trap density of CQDs exchanged using BA, 4-CH_3_-BA, and EDT, we measured dark currents of hole-only devices (HODs) with the structure of ITO/NiO_*x*_/PbS CQDs/MoO_3_/Au (Fig. 2e). The trap density is estimated from the trap-filled regime, while the hole mobility is estimated from the space charge-limited current (SCLC) regime (Supplementary Note 1). From the SCLC regime, the hole mobilities were estimated to be 1 × 10^−4^, 7 × 10^−5^, and 2 × 10^−4^ cm^−2^ V^−1^ s^−1^ for BA-, 4-CH_3_-BA-, and EDT-exchanged CQD films, respectively (Supplementary Table 3). As the ligand size decreases, the hole mobility of the CQD films increases, a finding we assign to the enhanced inter-dot coupling. From the trap-filled regime, we estimated the trap densities to be 5 × 10^16^, 5 × 10^16^, and 2 × 10^17^ cm^−3^ for the CQD films with BA, 4-CH_3_-BA, and EDT, respectively. The BA-functionalized HTLs fabricated with the CQD inks thus showed lower trap densities compared to the EDT-exchanged HTL fabricated via solid-state ligand exchange.

To compare the morphologies of the CQD films exchanged with BA, 4-CH_3_-BA and EDT, we carried out scanning electron microscopy (SEM) and atomic force microscopy (AFM) studies (Supplementary Fig. 4). The BA-functionalized CQD films fabricated using CQD inks exhibited continuous and smooth morphologies without cracks and pinholes. A root mean square roughness <1 nm was achieved for the BA-functionalized CQD films. In contrast, the EDT-exchanged CQD film showed a rough morphology with cracks. These cracks have previously been noted for CQD films fabricated via solid-state ligand exchange, a result of the considerable volume contraction that follows the exchange of the bulky OA ligands with small ligands^19–21^. The rough EDT-exchanged film may contribute to increased leakage currents in devices.

### Orthogonality between polar and weakly polar CQD inks

To fabricate the light-absorbing layer, we used halide-passivated CQDs dispersed in butylamine:dimethylformamide (DMF) (4:1 v/v). The use of an orthogonal solvent (chlorobenzene) to fabricate BA-functionalized HTLs purposes to prevent degradation of the underlying light-absorbing layer. Solid-state EDT-exchanged CQD films are obtained by deposition of OA-capped CQDs, soaking the OA-capped film in EDT/acetonitrile (ACN) solution, and washing away unbound ligands by spin-coating ACN. These procedures result in partial loss of ligands in the underlying light-absorbing layer^22,23,33^.

**Fig. 3:**
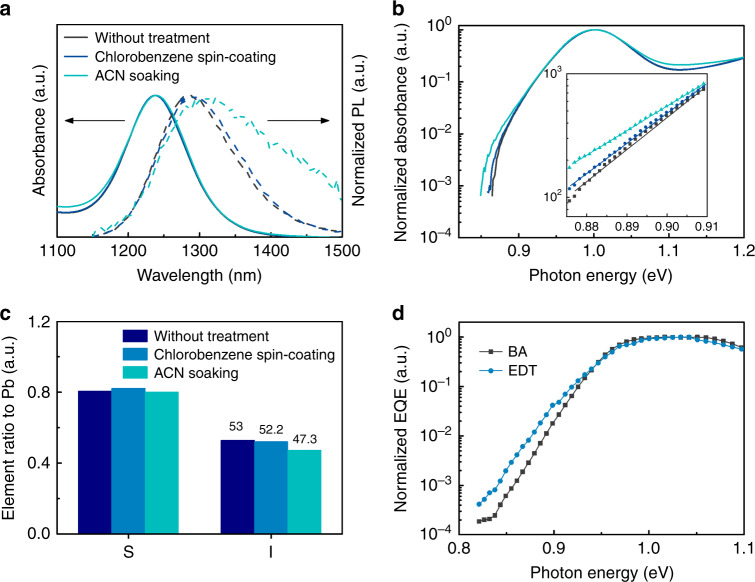
Degradation of light-absorbing layer upon the application of HTL-deposition protocols. **a** Normalized absorption and PL spectra of CQD films before and after solvent treatments. **b** Logarithmic-scale absorption spectra of CQD films to investigate bandtail absorption. **c** Element ratio-to-lead of CQD films before and after solvent treatments. **d** HDR EQE measurement of devices with BA HTL and EDT HTL to investigate the carrier distribution over the energy range below the bandgap.

To investigate the effect of the protocols for depositing HTLs on the underlying CQD layer, we measured absorption and PL spectra of the light-absorbing layers after spin-coating chlorobenzene (for BA-functionalized films) and soaking with ACN (for EDT film) (Fig. 3a). After we soaked using ACN, CQD films exhibited a red-shifted and broader PL spectrum, as well as a considerably weaker PL intensity compared to pristine CQD films (Fig. 3a and Supplementary Fig. 5), indicating the creation of defect states in the underlying CQDs. In contrast, the chlorobenzene-treated CQD films showed a similar PL intensity and spectrum compared to the pristine film, showing no remarkable change in optical and photophysical characteristics of the underlying CQDs.

The creation of localized trap states was investigated by observing the bandtail absorption of CQD films before and after solvent treatments (Fig. 3b)^26,34^. The chlorobenzene-treated CQD films showed unchanged bandtail absorption, whereas bandtail absorption was observed for the ACN-soaked film. To understand the origins of trap states in the CQD film following soaking with ACN, we performed X-ray photoelectron spectroscopy (XPS) to study the elemental compositions of CQD films. These revealed that the variation in the iodide to Pb ratio is negligible after spin-coating chlorobenzene, while the iodide ratio to Pb was reduced from 53 to 47% after soaking with ACN. We conclude that a portion of iodide ligands detached and washed away from the QD surface during soaking with ACN, leaving the CQD surface underpassivated. This we correlate with trap states arising due to incomplete passivation of the CQD surface upon the partial loss of iodide ligands.

To investigate energetic disorder in devices, we measured high dynamic range EQE (HDR EQE) for devices with the architecture of ITO/ZnO/IR-absorbing PbS CQD layer (*E*_*g*_ = 1.0 eV)/HTLs (*E*_*g*_ = 1.3 eV)/Au (Fig. 3d). This technique reports on the density of states in bandtail states^26,34^. Devices fabricated with BA HTL showed a steeper slope of the bandtail EQE than devices fabricated with EDT HTL.

### PV performance

**Fig. 4:**
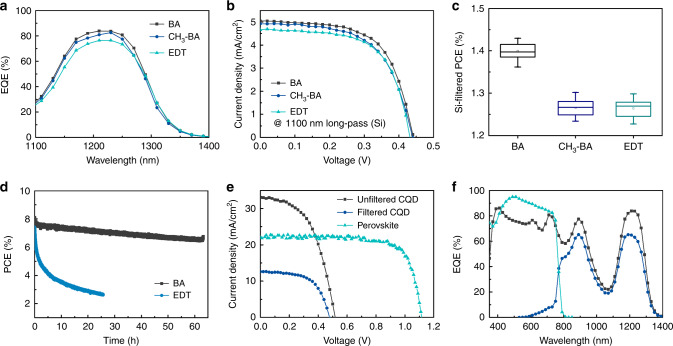
CQD PV performance. **a** *J–V* characteristics of CQD PVs with different HTLs under AM 1.5 illumination with 1100-nm cutoff silicon filter. **b** EQE spectra of CQD PVs with different HTLs beyond 1100 nm. **c** Statistics of device performance with different HTLs using 1100-nm cutoff silicon filter. **d** MPP stability of unencapsulated CQD PVs with BA HTL and EDT HTL under continuous 1-sun illumination and nitrogen flow. **e** *J–V* characteristics and **f** EQE spectra of perovskite front cell and CQD back cell with BA HTL in the 4T tandem configuration.

To test the performance of CQD PV devices employing different HTLs, we measured the EQE spectra and the current density–voltage (*J–V*) characteristics of PbS CQD PVs fabricated with BA, 4-CH_3_-BA, and EDT. We studied these under AM 1.5 illumination passed through a 1100-nm cutoff silicon filter (Fig. 4a, b). The CQD PVs with orthogonal CQD inks exhibited an EQE of 84% at the excitonic peak (1210 nm) and also a higher open-circuit voltage (*V*_OC_) than devices with EDT. A slightly lower fill factor (FF) was observed for devices with CH_3_-BA due to the lower hole mobility of the CH_3_-BA-excahnged layer. Si-filtered PCE values (the 1100-nm cutoff silicon filter) from more than 60 devices (20 devices for each ligand type) reveal that BA-based devices led to a statistically significant increase in PCE compared to devices with CH_3_-BA and EDT as seen in Fig. 4c. Specifically, the Si-filtered PCE was 1.4 ± 0.02% for BA devices compared to 1.28 ± 0.02% for CH3-BA and 1.28 ± 0.02% for EDT. The best PV performance with a record PCE of 1.43%, a *V*_OC_ of 0.438 V, a short-circuit current density (*J*_SC_) of 5.34 mA cm^−2^, and a FF of 61.3% was achieved for the BA-based device with the 1100-nm cutoff silicon filter (Supplementary Table 4). We then investigated the operating stability of unencapsulated devices fabricated with the BA HTL and the EDT HTL, investigating at the maximum power point (MPP) under AM 1.5G illumination and under nitrogen flow (Fig. 4d). BA-based devices showed considerably improved operational stability at MPP compared to EDT-based devices, retaining over 80% of initial PCE following continuous operation for 60 h under 1-sun illumination.

We then characterized the performance of 4T tandem cells by stacking BA-based CQD PV with a semitransparent perovskite front cell. The semitransparent perovskite front cell was prepared with the structure ITO/SnO_2_/perovskite (1.63 eV)/spiro-MeOTAD/MoO_3_/indium zinc oxide/MgF_2_. The semitransparent perovskite solar cell showed a reverse scan *J*_SC_ of 22.1 mA cm^−2^ and PCE of 18.5% (Supplementary Fig. 6). Figure 4d, f combines the 4T *J–V* characteristics and EQE spectra of each constituent cell in the 4T structure. BA-based CQD PV devices provide an additional *J*_*SC*_ of 12.6 mA cm^−2^ and PCE of 3.57% on top of the semitransparent perovskite solar cell. The PV parameters are summarized in Supplementary Table 4. The all-solution-processed perovskite/CQD tandems show excellent photon-harvesting capability, achieving a *J*_SC_ of 34.7 mA cm^−2^ and a PCE of 22%.

We then tested the performance of PbS CQD photodetectors, comparing the use of the BA HTL and EDT HTL with respect to dark *J–V* curve, specific detectivity (D^*^), and transient photocurrent (TPC) response (Supplementary Note 2 and Supplementary Fig. 7). The BA-based photodetectors showed lower dark current density and noise equivalent power compared to EDT-based photodetectors (Supplementary Fig. 7a). To characterize sensitivity, we measured D^*^ for the photodetectors with BA and EDT (Supplementary Fig. 7b). The BA-based photodetectors showed a higher peak D^*^ value of 1.4 × 10^12^ Jones at 1230 nm than the EDT-based photodetector (1.0 × 10^12^ Jones). The high D^*^ of the BA-based photodetector was achieved through the combination of high EQE and low noise.

## Discussion

In summary, we investigated the effect of ligand functional groups on the solubility of CQDs in weakly polar solvents. We employed aromatic ligands and embarked on a combination of experimental and computational studies. These revealed that the ligand dipole is a critical parameter for predicting the ink solubility. BA-functionalized CQDs are more soluble in chlorobenzene than BT-functionalized CQDs, a finding we assign to the stronger electrostatic potential of the BA ligand bound to the CQD surface. The development of weakly polar CQD inks enables smooth and crack-free HTL films that exhibit reduced trap density compared to conventional HTLs fabricated using solid-state ligand exchange. The chemical orthogonality of weakly polar CQD inks enables the fabrication of HTLs without degradation of the underlying light-absorbing layer, whereas the solid-state ligand exchange gives rise to trap states in the layer beneath. As a result, we fabricate the solid-state ligand exchange-free CQD PVs, achieving an EQE of 84% at 1210 nm. This results in addition of 1.43% and 3.57% extra efficiency on top of silicon and perovskite solar cells, respectively.

## Methods

### Materials

BA (99.5%), 4-CH_3_-BA (98%), BT (97%), 4-CH_3_-BT (98%), chlorobenzene (99.8%, anhydrous), butylamine (99.5%), DMF (99.8%, anhydrous), and hexane (99%) were purchased from Sigma-Aldrich and used without further purification. Lead iodide beads, lead bromide, and sodium acetate powders were purchased from Alfa Aesar.

### Synthesis of weakly polar CQD inks for HTLs

OA-capped CQDs were synthesized using a previously published method^35^. Orthogonal CQD inks for HTLs were prepared in a nitrogen-filled glovebox. The ligand solution was prepared by dissolving the functionalized aromatic ligands (0.3 M) in an appropriate solvent/solvent mixture (Supplementary Table 2). For BT-functionalized ligands, the same molar amount of triethylamine (TEA) was added in the ligand solutions to deprotonate BT-functionalized ligands. The addition of TEA improves the solubility of 4-CH_3_-BT-exchanged CQDs in chlorobenzene (Supplementary Fig. 8). In contrast, the BA ligands are readily deprotonated without the addition of TEA, a result of the stronger acidity than the OA. As a result, we did not observe a noticeable difference in the solubility of BA-functionalized CQDs upon the addition of TEA. One milliliter of the ligand solution was added dropwise to 1 mL of CQD solution (20 mg mL^*–*1^ in toluene) with stirring, and the solution was stirred for 10 min. 2.5 mL of hexane was added to precipitate the exchanged CQDs and then centrifuged at 7000 rpm for 2 min. For the purification of CQDs, the precipitated CQDs were dispersed in 1 mL of chlorobenzene and then 1.5 mL of hexane was added to precipitate the CQDs. To completely remove the OA byproduct and the residual ligands, the exchanged CQDs were washed with the purification steps three times.

### Synthesis of polar CQD inks for light-absorbing layers

The preparation of CQD inks for light-absorbing layer was carried out in air. The ligand solution was prepared by dissolving lead iodide (0.1 M), lead bromide (0.02 M), and sodium acetate (0.04 M) in DMF. A 5 mL of CQD solution dissolved in octane (6 mg mL^*–*1^) was added to 5 mL of ligand solution. After vigorously vortexing for 5 min, CQDs were transferred to DMF phase, and the DMF solution was washed with octane three times to remove residual OA ligands. The CQD solution was precipitated by adding toluene and dried in a vacuum. The CQDs were dispersed in butylamine:DMF (4:1 v/v).

### CQD PV fabrication

All fabrication processes were carried out in the air. The ZnO nanoparticles were synthesized using a published method^10^. The ZnO solution was spin-cast onto ITO substrate at 4000 rpm for 25 s. Then, the halide-passivated PbS CQDs as light-absorbing layer were deposited onto the ZnO/ITO substrate by dropping the CQD ink (300 mg mL^*–*1^) while the substrate is spinning at 800 rpm. Then, orthogonal CQD inks (20 mg mL^*–*1^) were spin-cast to fabricate HTLs at 2500 rpm for 25 s. For the EDT-exchanged HTL, OA-capped CQDs (25 mg mL^*–*1^) were spin-cast at 2500 rpm for 10 s, and soaked with a 0.01-vol.% EDT solution in ACN for 30 s, and then followed by three times of washing with ACN. Two EDT-exchanged layers were deposited to avoid direct contact between the light-absorbing layer and the top electrode due to numerous cracks in the EDT-exchanged layer. Finally, a 120-nm Au electrode was deposited by e-beam evaporation.

### CQD PV characterization

The active area (0.049 cm^2^) was determined by placing an aperture between the devices and the AM 1.5 solar simulator (Sciencetech class A). The *J–V* characteristics were measured using a Keithley 2400 source meter under simulated AM 1.5 illumination and a nitrogen atmosphere. The light intensity was calibrated using a reference solar cell (Newport). The *J–V* curves of CQD PVs were scanned from *−*0.70 to +0.1 V at 0.02-V interval steps without wait time between voltage steps. During the CQD:perovskite 4T measurement, CQD devices were masked by 0.049 cm^2^ of aperture and placed behind the semitransparent perovskite front cell.

A monochromatic illumination (400-W Xe lamp) with appropriate cutoff filters was used as an excitation source. For EQE calibration, Newport 818-UV and Newport 838-IR photodetectors were used. The response was measured at short-circuit conditions with a Lakeshore preamplifier feeding into a Stanford Research 830 lock-in amplifier. HDR EQE was measured at the sensitivity of the preamplifier of 1 nA V^*–*1^, ensuring the sufficient resolution of the EQEs at the IR region >1100 nm.

### CQD photodetector characterization

The average noise current was measured using multiple readings from a SR830 lock-in using its noise measurement system. The detector was connected with a low-noise SR570 preamplifier to the lock-in. The noise currents of photodetectors were measured at a frequency of 1 kHz under zero bias. For TPC measurement, femtosecond laser pulses (1030 nm, 5-kHz repetition rate) generated by a Yb:KGW laser (Pharos, Light Conversion), passed through an optical parametric amplifier (Orpheus, Light Conversion) selected for 600-nm wavelength. The signals were recorded using an oscilloscope with an input impedance of 50 Ω.

### DFT calculations

DFT calculations were performed using the Quickstep module of the CP2K software^36^. Goedecker–Teter–Hutter pseudopotentials^37^ within the generalized gradient approximation with Perdew–Burker–Ernzerhoff exchange-correlation functional^38^, a 600 Ry real space grid cutoff, DFT-D3 empirical van der Waals corrections^39^, double-zeta plus polarization basis set optimized in molecules^40^ were used in all calculations. A quantum dot of ~1 nm in diameter in a (32 Å)^3^ unit cell was prepared starting from bulk PbS geometry, terminated by Pb and capped by ligands in the amount to exactly charge-balance the excess of cations. Four chlorobenzene molecules were inserted into available empty spaces in the ligand shell with Cl oriented both toward and away from the QD and then fully relaxed. Solvent interaction energy was calculated as an average over these four molecules.

### Optical and morphological characterization

Steady-state and time-resolved PL measurements were carried out using a Horiba Fluorolog time-correlated single-photon counting setup, equipped with a photomultiplier tube detector. A monochromatized Xe lamp (*λ*_ex_ = 650 nm) and a pulsed diode laser (*λ*_ex_ = 723 nm) were used as excitation sources for steady-state and time-resolved PL, respectively. Optical absorption measurements were performed using a PerkinElmer Lambda 950 system. The film morphology was characterized using an Asylum Research Cypher AFM and a Hitachi SEM (SU8230).

### UPS, XPS, and FTIR measurements

UPS measurement was carried out using a helium discharge source (HeI α, *hv* = 21.22 eV). UPS spectra were obtained at a *−*5-V bias relative to the spectrometer to efficiently collect low kinetic-energy electrons. XPS measurement was performed using a spectrometer (Thermo Scientific) and a monochromatic K-Alpha X-ray system, with a 75-eV pass energy, and an energy steps of 0.05 eV. FTIR spectra were observed using a spectrometer (Thermo Scientific iS50) in a spectral range of 400–4000 cm^*–*1^ with ATR accessory.

### Reporting summary

Further information on research design is available in the Nature Research Reporting Summary linked to this article.

## Supplementary information

Supplementary Information

Reporting Summary

## Data Availability

The data that support the findings of this study are available from the corresponding authors upon reasonable request.
