# Supplementary material for: Orthogonal colloidal quantum dot inks enable efficient multilayer optoelectronic devices
Source: Nat Commun. 2020 Sep 23;11:4814. doi: 10.1038/s41467-020-18655-7 (PMC7511352; doi:10.1038/s41467-020-18655-7)
Supplement: Supplementary file 1 — Supplementary Information [file 41467_2020_18655_MOESM1_ESM.pdf]

Orthogonal Colloidal Quantum Dot Inks Enable Efficient Multilayer Optoelectronic Devices

Seungjin Lee[1†], Min-Jae Choi[1,3†], Geetu Sharma[2], Margherita Biondi[1], Bin Chen[1], Se-Woong Baek[1], Amin Morteza Najarian[1], Maral Vafaie[1], Joshua Wicks[1], Laxmi Kishore Sagar[1], Sjoerd Hoogland[1], F. Pelayo García de Arquer[1], Oleksandr Voznyy[2*] and Edward H. Sargent[1*]

[1]Department of Electrical and Computer Engineering, University of Toronto, 35 St George Street, Toronto, Ontario M5S 1A4, Canada.

[2]Department of Physical and Environmental Sciences, University of Toronto Scarborough, Scarborough, Ontario, M1C 1A4, Canada.

[3]Present address: Department of Chemical and Biochemical Engineering, Dongguk University, Seoul 04620, Republic of Korea

[†]These authors contributed equally to this work

[*]Correspondence email: ted.sargent@utoronto.ca; o.voznyy@utoronto.ca

Supplementary Note 1. Determination of trap densities and hole mobilities from HODs

A trap-filling region can be identified by significant increase of the current injection at a voltage (> V_{TFL}) where all the traps are filled. From this region, the trap density was estimated using following equation.

$$n_t = \frac{2V_{TFL}\varepsilon\varepsilon_0}{eL^2}$$

where V_{TFL} is the trap-filled limit voltage, ε is relative dielectric constant (18 for PbS CQDs)[1], ε_0 is the vacuum permittivity, e is the electron charge, and L is the thickness of the CQD films. For HODs, we fabricated thicker CQD films than the HTLs used in the PVs and PDs fabricated in PVs and PDs. We measured the thicknesses of HTLs by Dektak. The thicknesses of HTLs are 87, 92, and 83 nm, for BA-, 4-CH_3-BA-, and EDT-exchanged CQD films, respectively. The HODs with BA functionalized HTLs showed lower V_{TFL} (0.21 V) than the HOD with EDT-exchanged HTL (0.65 V). The trap densities were estimated to be 4.94×10^{16}, 5.52×10^{16}, and 1.88×10^{17} cm^{-3} for BA-, 4-CH_3-BA-, and EDT-exchanged CQD films, respectively.

Mobility calculation

A SCLC region can be identified by a slope of 2 in double logarithmic scale. In SCLC region, the current is dominated by charge carrier injected from the electrodes. The mobility was calculated using following equation.

$$J = \frac{9}{8}\varepsilon\varepsilon_0\mu\frac{V^2}{L^3}$$

where ε is relative dielectric constant (18 for PbS CQDs), ε_0 is the vacuum permittivity, L is the thickness, and μ is mobility. The mobilities were estimated to be 1.08×10^{-4}, 6.81×10^{-5}, and 2.10×10^{-4} cm^{-2} V^{-1} s^{-1} for BA-, 4-CH_3-BA-, and EDT-exchanged CQD films, respectively.

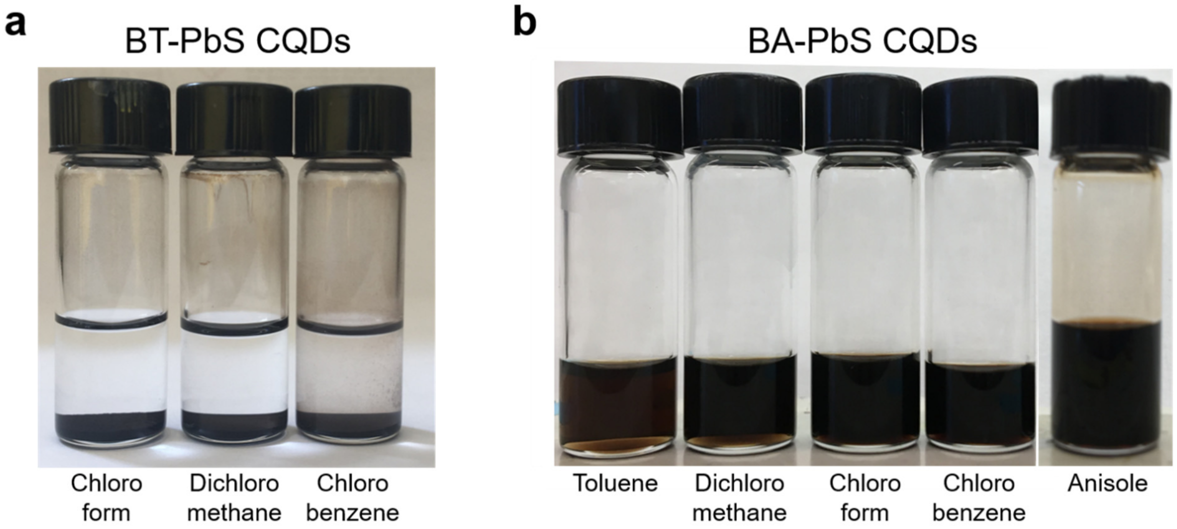

Supplementary Fig. 1 Photographs showing **a** BT-exchanged PbS CQDs, and **b** BA-exchanged PbS CQDs in various solvents.

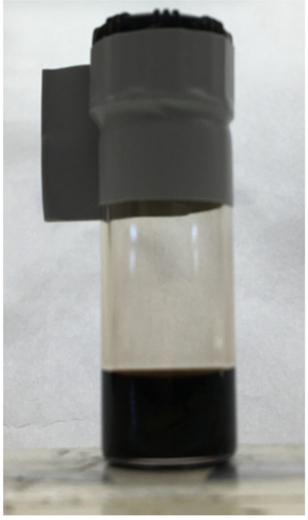

Supplementary Fig. 2 Photographs showing BA-exchanged PbS CQDs in chlorobenzene after 6 months in air.

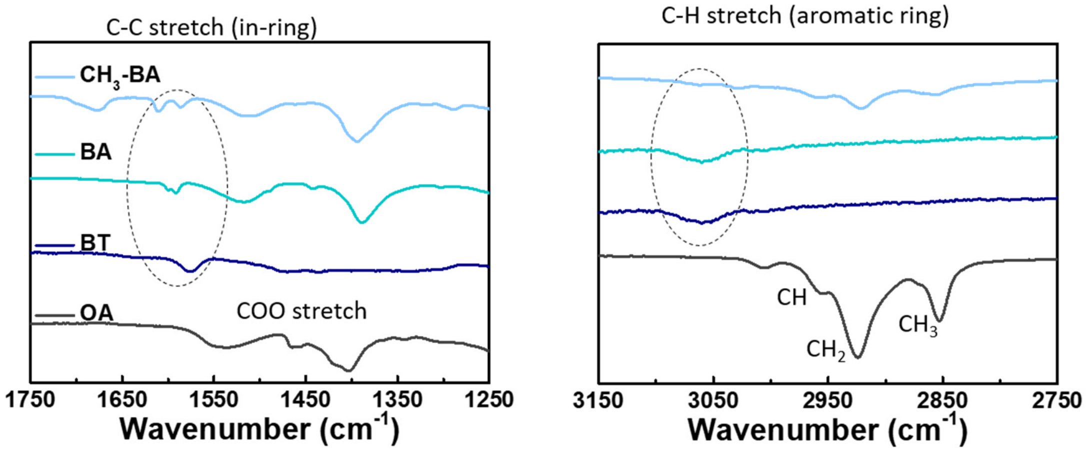

Supplementary Fig. 3 FTIR spectra of PbS CQD films with four different ligands.

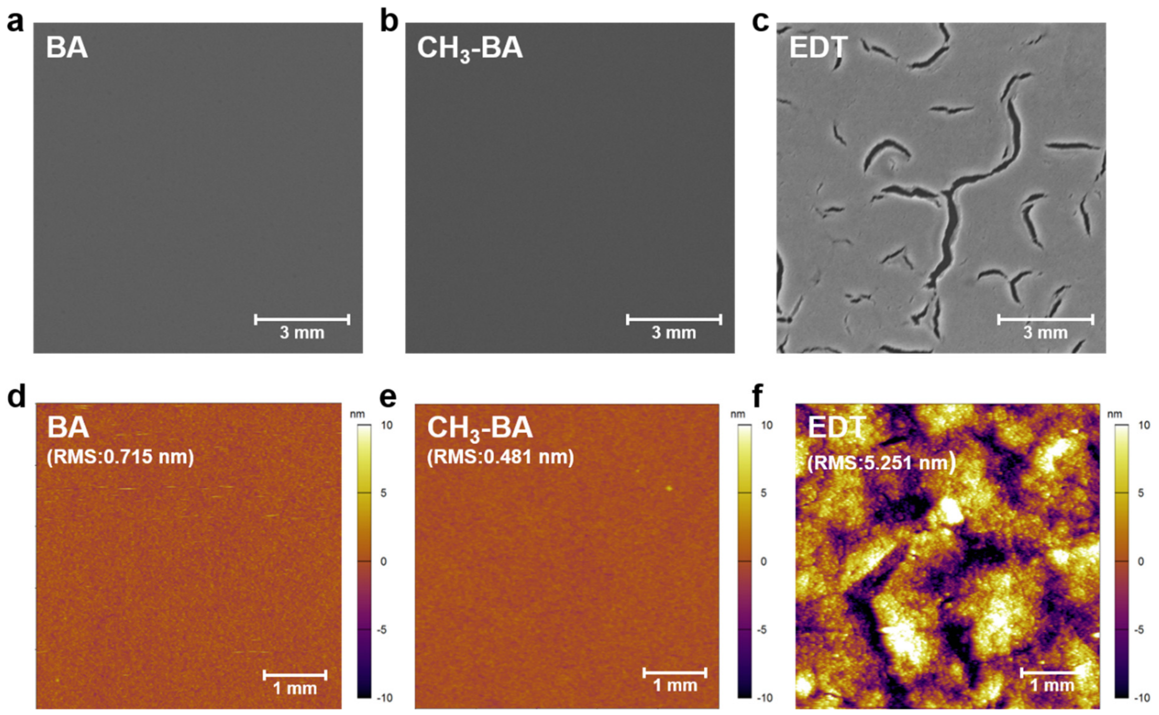

Supplementary Fig. 4 Morphology of HTLs prepared from different inks. SEM images of **a** BA-exchanged PbS CQD film, **b** CH_3-BA-exchanged PbS CQD film, and **c** EDT-exchanged CQD film by the SSE. AFM images of **d** BA-exchanged PbS CQD film, **e** CH_3-BA-exchanged PbS CQD film, and **f** solid-state EDT-exchanged CQD film.

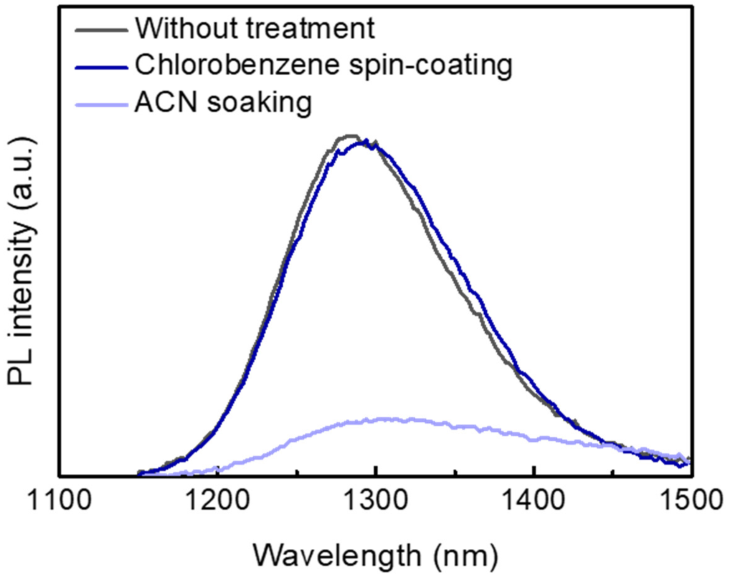

Supplementary Fig. 5 PL spectra of the PbS CQD films before and after solvent treatments.

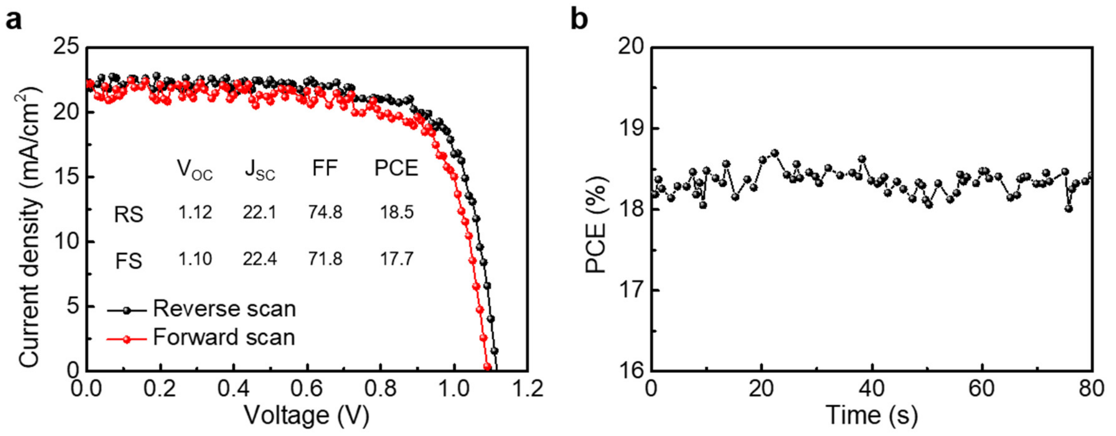

Supplementary Fig. 6 PV parameters for semi-transparent perovskite front cells. a Forward and reverse scan of semi-transparent perovskite front cells. **b** Short-term MPP tracking showing the stabilized PCE of the device.

Supplementary Note 2. Photodetector performance

We then analyzed the performance of PbS CQD photodetectors with BA HTL and EDT HTL through several key metrics: dark *J–V* curve, specific detectivity (D^*), and transient photocurrent (TPC) response. The devices showed typical diode *J–V* characteristics in the dark, displaying a saturation current under reverse bias (Supplementary Fig. 7a). The BA-based photodetector showed lower dark current density (52 nA/cm^2) at –1 V compared to the EDT-based photodetector (180 nA/cm^2). The low dark current for the BA-based device may be attributed to the smooth and crack-free BA HTL as well as the suppression of trap formation in the light absorbing layer underneath. The noise equivalent power (NEP) was then measured to be 1.77×10^{-13} and 2.30×10^{-13} W Hz$^{-1/2}$ at a frequency of 1 kHz for the photodetectors with BA and EDT, respectively. To characterize the sensitivity of the photodetectors, we measured the D^* spectra for the photodetectors with BA and EDT (Supplementary Fig. 7b). The BA-based photodetector showed a higher peak D^* value of 1.4×10^{12} Jones at 1230 nm than the EDT-based photodetector (9.9×10^{11} Jones). The high D^* of the BA-based photodetector was achieved through the combination of high EQE and low NEP.

The response speed is another significant parameter for high-speed photodetector applications. TPC measurements were performed using a 600 nm femtosecond pulsed laser in order to explore charge transport and collection in the photodetectors with different HTLs. By defining the falling time as the times taken by the signal to decrease from 90% to 10%[2], the fall time of the BA and EDT devices with an active area of 0.03 mm^2 is measured to be 240 and 335 ns, respectively (Supplementary Fig. 7c). Although the mobility of BA HTL is lower than that of EDT HTL, the BA-based photodetector showed faster response time than the EDT-based photodetector. A possible reason is that the transit time of charges through the HTL may not be the key determinant

of the response since the HTL is significantly thinner (20–40 nm) compared to the light absorbing layer (~500 nm)[3]. Instead, the transit time of charges within the light absorbing layer or the charge collection time at interfaces between the charge transport layer and the light absorbing layer can determine the response time of the photodetectors. The larger amount of trap states in the EDT HTL and the creation of trap states in the light absorbing layers lead to a larger amount of trap-mediated recombination in the EDT-based photodetector, retarding charge transport through the light absorbing layer and charge collection at the EDT HTL/light absorbing layer interface. We therefore attribute the faster response time of the BA-based photodetector to the reduced trap-mediated recombination in the device.

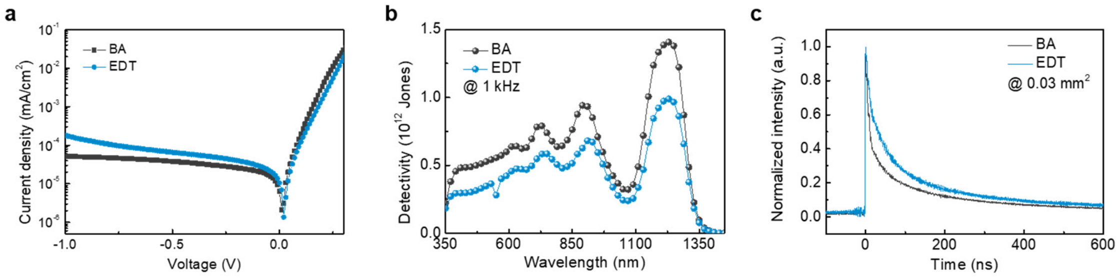

Supplementary Fig. 7 CQD photodetector performance. **a** *J-V* characteristics of CQD photodetectors with BA HTL and EDT HTL under dark condition. **b** Detectivity of CQD photodetectors with BA HTL and EDT HTL at a frequency of 1 kHz under zero bias. **c** TPC response of the photodetectors with active area of 0.03 mm^2.

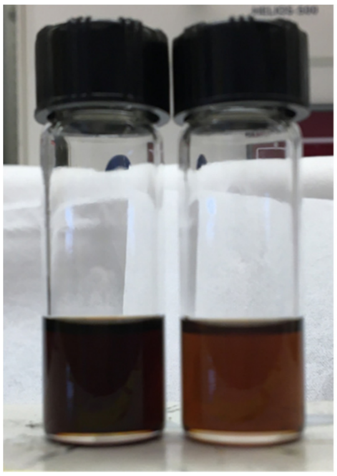

Supplementary Fig. 8 Photographs showing 4-CH_3-BT exchanged PbS CQDs with and without addition TEA during the ligand exchange.

Supplementary Table 1. The interaction energies of four different ligand-exchanged CQDs with chlorobenzene.

Ligand & CQDs	Interaction energy (eV)
BT & PbS	0.27
CH_3-BT & PbS	0.31
BA & PbS	0.50
CH_3-BA & PbS	0.69

Supplementary Table 2. Ligand exchange and purification details.

Ligand & CQDs	Ligand solvent	Antisolvent	Final solvent
BT·TEA & PbS	Toluene	Hexane	-
CH_3-BT·TEA & PbS	Toluene	Hexane	Chlorobenzene
BA & PbS	Toluene	Hexane	Chlorobenzene
CH_3-BA & PbS	Toluene	Hexane	Chlorobenzene
BA & InP/ZnSe/ZnS	Toluene:Isopropanol (2:1)	Hexane	Chlorobenzene
BA & InAs	Toluene:Isopropanol (2:1)	Hexane	Chlorobenzene

Supplementary Table 3. Summarized mobility and trap density of HTLs.

Device configuration	Mobility (cm^{-2} V^{-1} s^{-1})	Trap density (cm^{-3})
ITO/NiO_x/BA/MoO_3/Au	1.0×10^{-4}	4.9×10^{16}
ITO/NiO_x/CH_3-BA/MoO_3/Au	7.0×10^{-5}	5.5×10^{16}
ITO/NiO_x/EDT/MoO_3/Au	2.1×10^{-4}	1.9×10^{17}

Supplementary Table 4. PV parameters of CQD PVs with different HTLs using 1100 nm cut-off silicon filter, CQD back cell with BA HTL, and semi-transparent perovskite front cell.

Device	Filter type	J_{SC} (mA/cm^2)	V_{OC} (V)	FF	η (%)
BA	Solar spectrum longer than 1100 nm	5.34	0.438	0.613	1.43
CH_3-BA	Solar spectrum longer than 1100 nm	5.11	0.441	0.575	1.29
EDT	Solar spectrum longer than 1100 nm	4.87	0.430	0.619	1.30
BA	Without filter	33.12	0.518	0.517	8.87
BA	Perovskite filter	12.62	0.478	0.591	3.57
Semi-transparent perovskite front cell		22.12	1.118	0.748	18.5
Perovskite-CQD 4T tandem cell					22.07

Supplementary References

1 Speirs, M. J. *et al.* Temperature dependent behaviour of lead sulfide quantum dot solar cells and films. *Energy & Environ. Sci.* **9,** 2916-2924 (2016).

2 Wang, H. & Kim, D. H. Perovskite-based photodetectors: materials and devices. *Chem. Soc. Rev.* **46,** 5204-5236 (2017).

3 Dou, L. *et al.* Solution-processed hybrid perovskite photodetectors with high detectivity. *Nat. Commun.* **5,** 5404 (2014).
