# Supplementary material for: Orthogonal colloidal quantum dot inks enable efficient multilayer optoelectronic devices
Source: Nat Commun. 2020 Sep 23;11:4814. doi: 10.1038/s41467-020-18655-7 (PMC7511352; doi:10.1038/s41467-020-18655-7)
Supplement: Supplementary file 2 — Reporting Summary [file 41467_2020_18655_MOESM2_ESM.pdf]

nature research

Corresponding author(s): Edward H. Sargent

Solar Cells Reporting Summary

Nature Research wishes to improve the reproducibility of the work that we publish. This form is intended for publication with all accepted papers reporting the characterization of photovoltaic devices and provides structure for consistency and transparency in reporting. Some list items might not apply to an individual manuscript, but all fields must be completed for clarity.

For further information on Nature Research policies, including our data availability policy, see Authors & Referees.

Experimental design

Please check: are the following details reported in the manuscript?

1. Dimensions

Item	Yes	No	Details
Area of the tested solar cells	☒	☐	Described in Methods (PV characterization).
Method used to determine the device area	☒	☐	Described in Methods (PV characterization). Optical aperture is used to define the active area.

2. Current-voltage characterization

Item	Yes	No	Details
Current density-voltage (J-V) plots in both forward and backward direction	☒	☐	For pereovskite devices, forward and reverse scan are shown in Supplementary Fig. 5. For CQD devices, no hysteresis was observed for all CQD PV devices, including both the EDT devices and BA devices. The hysteresis-free behavior in CQD PVs is also widely-accepted in literatures.
Voltage scan conditions<br>*For instance: scan direction, speed, dwell times*	☒	☐	Described in Methods (PV characterization).
Test environment<br>*For instance: characterization temperature, in air or in glove box*	☒	☐	Described in Methods (PV characterization). Devices were tested in N2. Temperature 25c
Protocol for preconditioning of the device before its characterization	☒	☐	Described in Methods (PV characterization).
Stability of the J-V characteristic<br>*Verified with time evolution of the maximum power point or with the photocurrent at maximum power point; see ref. 7 for details.*	☒	☐	For perovskite devices, maximum power point tracking is shown in Supplementary Fig. 5b.

3. Hysteresis or any other unusual behaviour

Item	Yes	No	Details
Description of the unusual behaviour observed during the characterization	☐	☒	No hysteresis was observed for all CQD PV devices, including both the EDT devices and BA devices. The hysteresis-free behavior in CQD PVs is also widely-accepted in literatures.
Related experimental data	☒	☐	For perovskite devices, Forward and reverse scan are shown in Supplementary Fig. 5a.

4. Efficiency

Item	Yes	No	Details
External quantum efficiency (EQE) or incident photons to current efficiency (IPCE)	☒	☐	EQE data is described in Fig. 4.
A comparison between the integrated response under the standard reference spectrum and the response measure under the simulator	☒	☐	Described in the manuscript (Fig. 4).
For tandem solar cells, the bias illumination and bias voltage used for each subcell	☒	☐	This work is for 4T tandem (PV characterization) .

5. Calibration

Item	Yes	No	Details
Light source and reference cell or sensor used for the characterization	☒	☐	Newport, Class A simulator is used for the measurements (Methods, PV characterization).

Confirmation that the reference cell was calibrated and certified

☒ Yes ☐ No

The light intensity was calibrated by reference solar cell by Newport (Methods, PV characterization).

Calculation of spectral mismatch between the reference cell and the devices under test

☒ Yes ☐ No

Described in Methods (PV characterization).

6. Mask/aperture

Size of the mask/aperture used during testing

☒ Yes ☐ No

0.049 cm2 aperture. Described in Methods (PV characterization).

Variation of the measured short-circuit current density with the mask/aperture area

☒ Yes ☐ No

Described in Methods (PV characterization). Tested with aperture between solar source and devices.

7. Performance certification

Identity of the independent certification laboratory that confirmed the photovoltaic performance

☐ Yes ☒ No

We did not certify our cells.

A copy of any certificate(s)
Provide in Supplementary Information

☐ Yes ☒ No

We did not certify our cells.

8. Statistics

Number of solar cells tested

☒ Yes ☐ No

Total 60 devices were used for statistics described in Fig. 4c.

Statistical analysis of the device performance

☒ Yes ☐ No

Variation of device performance are shown in Fig. 4c.

9. Long-term stability analysis

Type of analysis, bias conditions and environmental conditions
For instance: illumination type, temperature, atmosphere humidity, encapsulation method, preconditioning temperature

☒ Yes ☐ No

The stability test at MPP operation conditions under AM 1.5G simulated illumination and nitrogen flow was carried out for unencapsulated solar cells (Fig. 4d)
